# Efficacy and safety of oral semaglutide in older patients with type 2 diabetes: a retrospective observational study (the OTARU-SEMA study)

**DOI:** 10.1186/s12902-024-01658-6

**Published:** 2024-07-24

**Authors:** Yuki Oe, Hiroshi Nomoto, Kyu Yong Cho, Kei Yokozeki, Tsubasa Ono, Aika Miya, Hiraku Kameda, Akinobu Nakamura, Yoshiaki Arimura, Tatsuya Atsumi

**Affiliations:** 1 Department of Rheumatology, Endocrinology and Nephrology, Faculty of Medicine and Graduate School of Medicine, Hokkaido University Graduate School of Medicine, North-15, West-7, Kita-ku, Sapporo, Hokkaido 060-8638 Japan; 2 Department of Diabetes, Otaru General Hospital, Wakamatsu-1-1-1, Otaru, Hokkaido 047-0017 Japan; 3 Division of Endocrinology, Metabolism, and Rheumatology, Department of Internal Medicine, Asahikawa Medical University, 2-1-1-1, Midorigaoka-Higashi, Asahikawa-City, Hokkaido 078-8510 Japan; 4 Department of Gastroenterology, Otaru General Hospital, Wakamatsu-1-1-1, Otaru, Hokkaido 047-0017 Japan

**Keywords:** Glucagon-like peptide 1 receptor, Aged, Type 2 diabetes, Cognitive test, Oral medicine

## Abstract

**Background:**

Oral semaglutide in older subjects with type 2 diabetes was as effective as in younger subjects, according to phase 3 clinical trials. However, its efficacy can be limited in very aged population, due to the presence of impaired cognitive function and the complex instructions for its use. Here, we investigated its efficacy and safety by further age bracket in older subjects in real-world.

**Methods:**

We retrospectively studied subjects > 65 years of age with type 2 diabetes who started oral semaglutide treatment. The primary outcome was the change in glycated hemoglobin (HbA1c) over 6 months. Adverse events and cognitive function were evaluated using the Common Terminology Criteria for Adverse Events (CTCAE) and the Hasegawa Dementia Rating Scale-revised (HDS-R). The achievement rate of glycemic targets was evaluated based on the age, health status of subjects and their use of anti-diabetic agents which can cause hypoglycemia, with additional analysis between two subgroups; early (65–74) versus late (≥ 75) older. Furthermore, we evaluated the relationships between their improvements in HbA1c and the baseline characteristics of the subjects, including their cognitive function and insulin secretory capacity.

**Results:**

We studied the efficacy of the drug in 24 subjects. Their HbA1c and body weight significantly decreased (− 13.1 ± 7.5 mmol/mol and − 3.0 ± 2.4 kg, respectively; *P* < 0.01). Although cognitive function was lower in the late older group (*r* = −0.57, *P* < 0.01), changes in HbA1c showed no difference between the two subgroups (*P* = 0.66) and it correlated with the insulin secretory capacity rather than cognitive function (*r* = −0.49, *P* < 0.05). Glycemic targets were more likely to be achieved (*P* < 0.01), but HbA1c excessively decreased in late older subjects who were also using insulin or an insulin secretagogue. The frequency of adverse events was similar to that in the clinical trial, whereas discontinuation of medication were more frequent among the late older subjects (Early; *n* = 2, Late; *n* = 4).

**Conclusions:**

Oral semaglutide improves the glycemic control of older subjects, but it might be a risk for potential hypoglycemia and discontinuation because of adverse events in subjects of ≥ 75 years. Attention should be paid to insulin secretory capacity and concomitant medications rather than concern about adherence.

**Supplementary Information:**

The online version contains supplementary material available at 10.1186/s12902-024-01658-6.

## Background

In aging societies, the prevalence of cognitive impairment is increasing and the risks and progression of disease are also increasing, owing to declines in biological functions [1–3]. Japan is now regarded as a super-aged society, and the population of patients with type 2 diabetes (T2D) is aging as well during last decade [4]. The incidence of diabetes complications is more frequent in older patients; therefore, glycemic management is of great importance in this population [5, 6]. However, some anti-hyperglycemic agents can cause severe hypoglycemia and are associated with the deterioration of arteriosclerosis and cognitive impairment [7, 8]. The avoidance of such adverse events and the maintenance of appropriate glycemic control should ensure better health and life expectancy.

Glucagon-like peptide-1 receptor agonists (GLP-1RAs) are anti-hyperglycemic agents delivering glucose lowering effects with low risk of hypoglycemia due to their glucose level-dependent mechanism [9]. A number of large-scaled trials have demonstrated the benefits of GLP-1RAs, notably with respect to the reduction in the incidence of cardiovascular events induced and the preservation of kidney function [10, 11]. However, only injectable products had been available until recent years. Thereby, patients who had difficulty in self-administering or feeling resistance to injections abandon such intensive treatments, despite its efficacy. Recently available oral semaglutide was the first peroral GLP-1RAs in the world. Although a large-scaled trial showed significant reduction of total or cardiovascular death [12], there are several limitations to its use. It must be taken on an empty stomach before first meal or beverage, with no more than 120 mL plain water only, and the ingestion of food, beverages, and other oral medications must be avoided for at least 30 min after taking it [13]. Such burden might prevent sufficient effectiveness especially in older patients, who may have cognitive impairment. Therefore, it is important to assess the efficacy of oral semaglutide in older subjects. However, this has only been assessed through a *post-hoc* exploratory analysis of a few clinical trials to date [14].

The purpose of this study was to evaluate the efficacy and safety of oral semaglutide in a real-world clinical setting focusing exclusively on older subjects. In addition, we aimed to characterize the cognitive function and baseline characteristics of older subjects that are associated with the efficacy of semaglutide treatment.

## Methods

### Study design and participants

We conducted a retrospective, single-arm, observational study (the OTARU-SEMA study). Older Japanese subjects with T2D who attended the Department of Diabetes, Otaru General Hospital, were recruited between December 2021 and August 2022. The eligible subjects were aged ≥ 65 years, had a glycated hemoglobin (HbA1c) level ≥ 53.0 mmol/mol (7%), and judged by in-hospital interviews to be able to manage their medications themselves; purchase and prepare their own medications, or at least take them on their own. Subjects who commenced the administration of oral semaglutide were followed for 6 months. The dosage was adjusted by the physician in charge and was increased as much as the subjects could tolerate. We excluded subjects who had previously been administering a GLP-1RA subcutaneously. The other exclusion criteria described in Supporting information.

The study was registered with the University Hospital Medical Information Network (UMIN) (registration number; UMIN 000048782, registration date; 29^th^ August 2022). It was approved by the Institutional Clinical Research Review Board of Otaru General Hospital (approval number 04-001) and was conducted in accordance with the principles of the Declaration of Helsinki and its amendments. An opt-out informed consent approach was adopted for all the participants.

### Adverse events

The presence and severity of adverse events were evaluated using the Common Terminology Criteria for Adverse Events (CTCAE), and the definitions of severe adverse events are provided in the Supporting information. In accordance with the instructions of the manufacturer, the participants were thoroughly informed regarding the known adverse events of GLP-1RAs, including gastrointestinal events and anorexia, before the administration. The administration procedure was explained by the physician in charge and a nurse, and compliance was confirmed at each hospital visit as part of standard clinical practice in Japan.

### Biochemical analyses and cognitive function testing

The body weight and height of subjects were measured using a calibrated scale. Body mass index (BMI) was calculated as body weight (kg) divided by the square of height (m^2^). Endogenous insulin secretion was evaluated by the measurement of C-peptide (CPR) and C-peptide index (CPI) [15], and the biochemical parameters, including plasma glucose, were measured in blood samples collected after an overnight fast. CPI was calculated as CPI = 100 × fasting CPR (ng/mL)/plasma glucose (mg/dL). Other parameters were measured using standard techniques, and other data, including the medical history of the participants, were collected by the attending physicians.

The cognitive function of the participants was evaluated using the Dementia Assessment Sheet for Community-based Integrated Care System 8-items (DASC-8) and the Hasegawa Dementia Rating Scale-revised (HDS-R) during the study period. The criteria for a diagnosis of impaired cognitive function using these screening tests were those previously defined [16, 17]. These questionnaires were completed by a single well-trained nurse in the consulting rooms.

### Outcomes and data analysis

The primary outcome was the change in HbA1c over the 6 months of oral semaglutide treatment. The secondary outcomes were the safety of semaglutide treatment and the changes in body weight and other biochemical data. The date on which semaglutide treatment was initiated was regarded as the baseline. As Visit − 1, HbA1c and body weight were first measured 1–3 months prior to baseline to exclude the possibility that these parameters were affected by prior treatments to semaglutide administration. The achievement of glycemic targets was evaluated using the Clinical Practice Guidelines of the Japan Diabetes Society (JDS) and the Japan Geriatrics Society (JGS) Joint Committee [18], based on the age, health status of subjects and their use of anti-diabetic agents which can cause hypoglycemia. Their health status was assessed using DASC-8 and they were allocated to three groups on this basis: normal, mildly impaired, and moderately impaired or above [18]. On the basis of the above guideline, the glycemic target range was defined as follows: With insulin or insulin secretagogues: Normal health status and 65–74 years, 47.5–57.4 mmol/mol; Normal health status and ≥ 75 years, 53.0–63.9 mmol/mol; Mildly impaired, 53.0–63.9 mmol/mol; Moderately impaired or above, 58.5–69.4 mmol/mol, Without insulin or insulin secretagogues: Normal health status or Mildly impaired in any age, 47.5–57.4 mmol/mol; Moderately impaired or above in any age, 58.5–69.4 mmol/mol. We performed a sub-analysis of the data by dividing participants into two subgroups: early (65–74 years old) and late (≥ 75 years old) older group [19]. In addition, we evaluated the relationships between the baseline characteristics of the subjects and their improvements in HbA1c, and the relationships of cognitive function test scores with each parameter.

Normally distributed data are expressed as mean ± SD and other data are expressed as median (interquartile range). Data were analyzed using JMP Pro 17.0.0 (SAS Inc., Cary, NC, USA) or GraphPad Prism 8.4.2 (GraphPad Software, Inc. San Diego, CA, USA). For before-and-after comparisons, Student’s *t*-test was used to analyze parametric data and Wilcoxon’s signed-rank test was used for non-parametric data. Fisher’s exact test was used to compare the numbers of subjects who achieved their glycemic targets. Parameters and their changes were compared between the age groups using the unpaired *t*-test. Pearson’s correlation coefficient was used to identify associations between parameters. All the tests performed were two-sided, and *P* < 0.05 was considered to represent statistical significance. The post hoc power calculation was performed using GPower® version 3.1.9.2. *P* < 0.05 indicated statistical significance.

## Results

### Characteristics of subjects

**Fig. 1:**
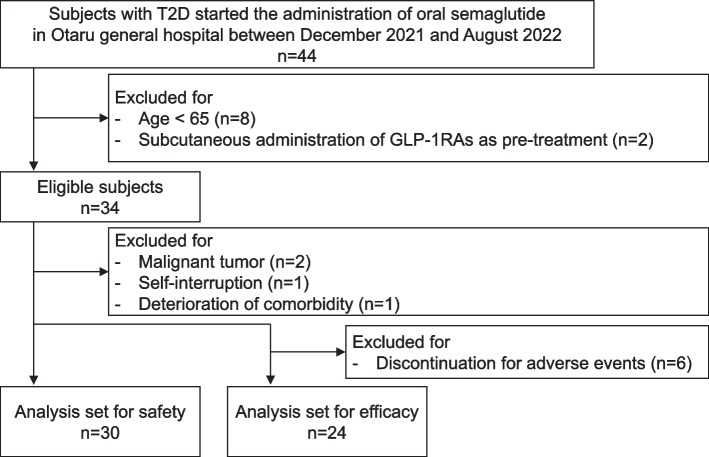
Flowchart of subject’s enrollment. The safety of the therapy was analyzed as a first cohort for 30 subjects and its efficacy was analyzed as a second cohort for 24 subjects that completed the study

Subjects were enrolled as described in Fig. 1. During the observation period, 44 subjects with T2D were administered oral semaglutide, but at the time of analysis, 10 were excluded because they were < 65 years (*n* = 8) or had previously subcutaneously administered a GLP-1RA (*n* = 2). Thus, 34 of the subjects were eligible for inclusion; however, a further 4 were excluded for the following reasons, unrelated to the drug: presence of a malignant tumor (*n* = 2), self-interruption (*n* = 1), and deterioration of a comorbidity (*n* = 1). Details of the adverse events and the reasons for the discontinuation of semaglutide were analyzed in the remaining 30 subjects as a safety analysis. Six of the subjects discontinued the medication, and therefore the remaining 24 subjects were studied with respect to efficacy.

Table 1 showed the baseline characteristics of the participants in an efficacy analysis. The prevalence of obesity (BMI ≥ 25 kg/m^2^) was 33.3% (Early; *n* = 5, Late; *n* = 3), but their insulin secretory capacity was preserved. In evaluation of cognitive function, the DASC-8 and HDS-R scores were consistent with cognitive impairment in only 4 (Early; *n* = 1, Late; *n* = 3) and 2 (Early; *n* = 0, Late; *n* = 2) subjects, respectively, and the others were not found to have impaired cognitive function. The final doses of semaglutide were as follows: 3 mg, *n* = 5 (Early; *n* = 4, Late; *n* = 1); 7 mg, *n* = 15 (Early; *n* = 8, Late; *n* = 7); and 14 mg, *n* = 4 (Early; *n* = 1, Late; *n* = 3). Few drugs were being used concomitantly; dipeptidyl peptidase-4 inhibitors (DPP-4is) were the most commonly prescribed drugs prior to semaglutide administration (Early; *n* = ,11 Late; *n* = 11), but all of the users were switched to oral semaglutide. Half of the subjects were being treated with anti-hyperglycemic agents that could be associated with severe hypoglycemia (sulfonylureas, glinides, and insulin), but only 1 subject who was being treated with a glinide showed severe renal dysfunction (eGFR ≤ 30 mL/min/1.73m^2^).

**Table 1:** Baseline characteristics

Variables	Analysis for	
	Efficacy (*N* = 24)	Safety (*N* = 30)
Age (years)	75.5 ± 6.9	76.2 ± 7.3
Female sex (n)	12	15
Height (cm)	155.2 ± 8.7	154.9 ± 9.1
Body weight (kg)	57.4 ± 11.5	56.4 ± 10.8
BMI (kg/m^2^)	23.7 ± 3.4	23.4 ± 3.1
Diabetes duration (years)^a^	17.0 [7.0, 23.0]	17.0 [9.5, 24.0]
FPG (mmol/L)^a^	7.7 ± 2.0	8.1 ± 2.2
HbA1c (mmol/mol)	63.1 ± 8.5	63.6 ± 8.0
CPR (nmol/L)^a^	0.6 ± 0.3	0.6 ± 0.3
CPI (ng/mL per mg/dL)^a^	1.5 ± 0.8	1.4 ± 0.8
Evaluation for cognitive function		
DASC-8	8.5 [8.0, 9.0]	9.0 [8.0, 9.0]
HDS-R	25.6 ± 3.8	25.4 ± 4.2
The proportion of anti-diabetic agent (n, %)		
The number of drugs	2.0 [2.0, 3.0]	2.0 [2.0, 3.0]
Biguanide	12 (50.0)	13 (43.3)
DPP-4i	22 (91.7)	28 (93.3)
SU	6 (25.0)	8 (26.7)
Glinides	4 (16.7)	5 (16.7)
Thiazolidine	1 (4.2)	1 (3.3)
α-GI	1 (4.2)	3 (10.0)
SGLT2i	11 (45.8)	14 (46.7)
Insulin	2 (8.3)	3 (10.0)

Values are expressed as mean ± SD or median (interquartile range). Data are shown for 24 subjects in an efficacy analysis and 30 subjects in a safety analysis

*BMI* Body mass index, *CPI* C-peptide index, *CPR* C-peptide, *DASC-8* Dementia assessment sheet for community-based integrated care system 8-items, *DPP-4i* Dipeptidyl peptidase-4 inhibitor, *FPG* Fasting plasma glucose, *HbA1c* Glycated hemoglobin, *HDS-R* Hasegawa dementia rating scale-revised, *SGLT2i* Sodium-glucose cotransporter 2 inhibitor, *SU* Sulfonylurea

^a^The values were calculated except one subject for missing data. α-GI, alpha glucosidase inhibitors

### Efficacy

**Fig. 2:**
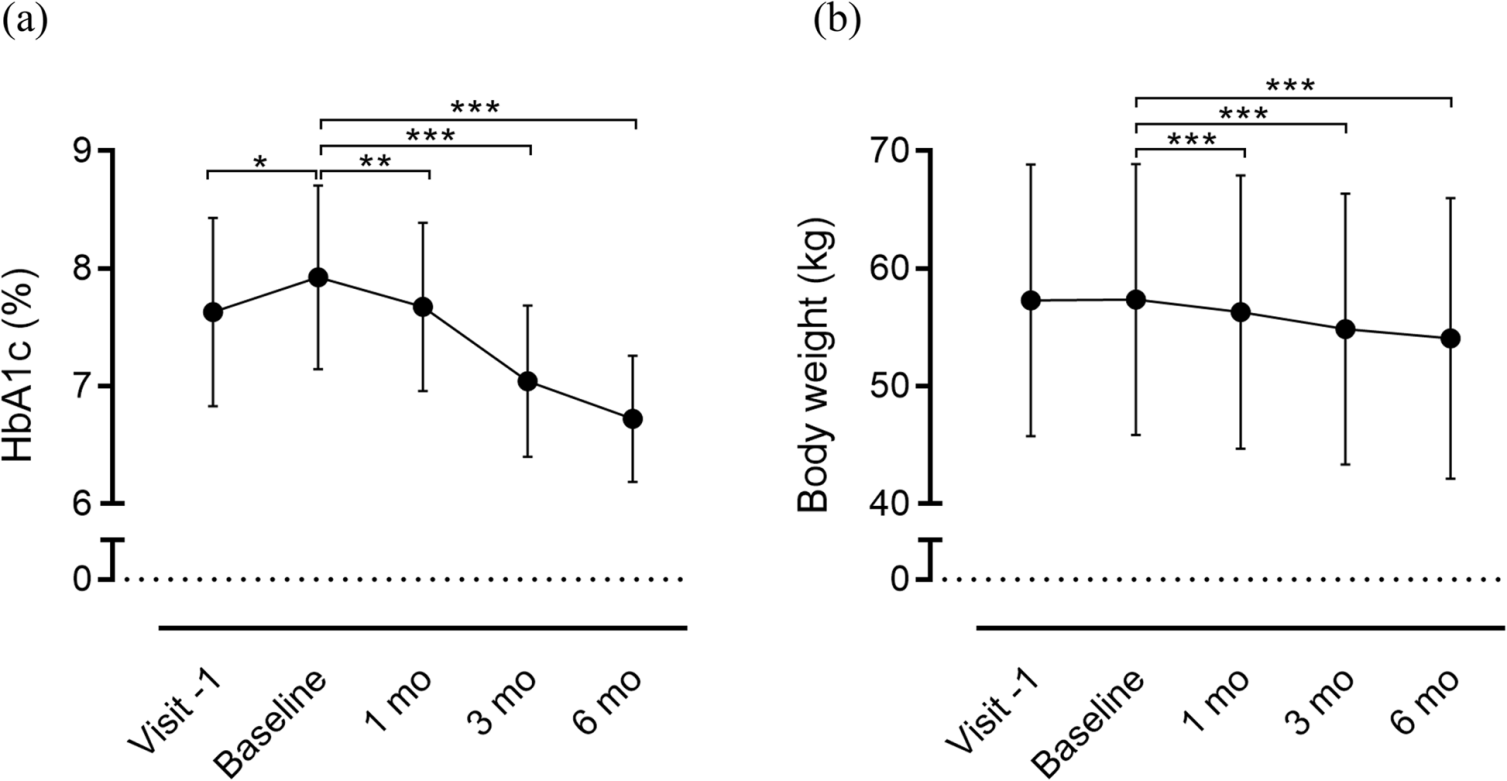
Outcomes after 6 months of therapy. **a** HbA1c, **b** Body weight. Both parameters showed significant reductions over the 6 months of the study. At baseline, the HbA1c levels were significantly higher and body weight did not differ in comparison to Visit −1. These changes were evaluated using Student’s *t*-test *versus* the beginning of administration. Values are mean ± SD. ** P < 0.01

**Fig. 3:**
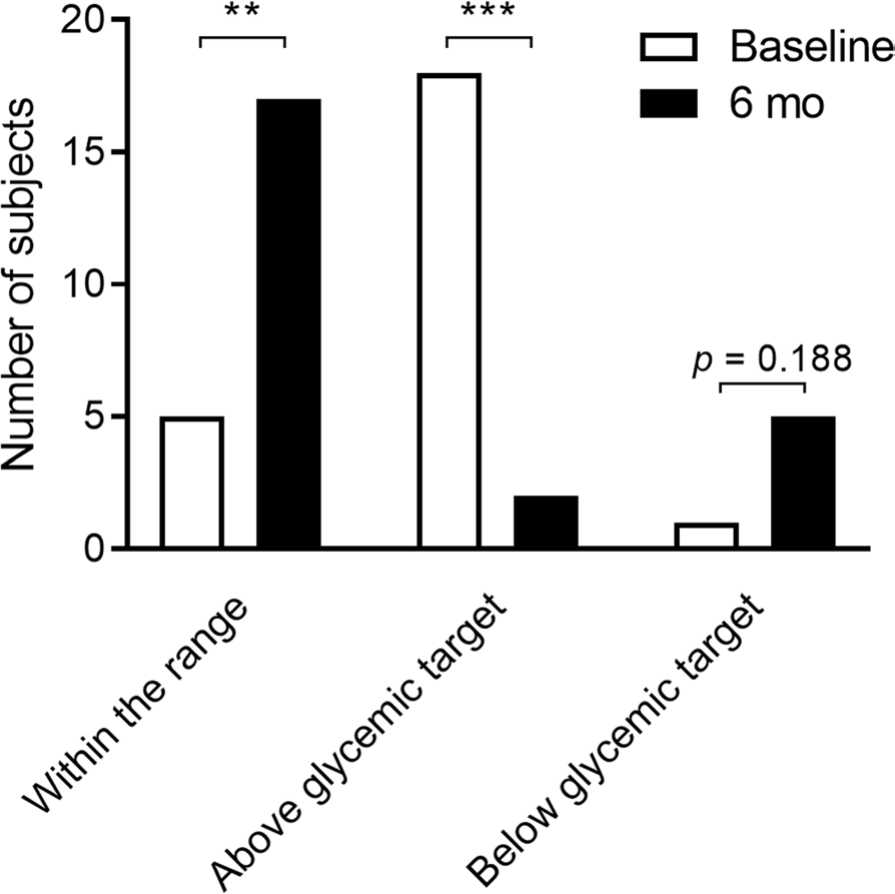
Change in the glycemic control of the participants. HbA1c was evaluated according to the clinical practice guidelines of the Japan Diabetes Society and the Japan Geriatrics Society Joint Committee. The glycemic target was set according to the baseline parameters, health status, and concomitant drug use of each subject. Fisher’s exact test was used to compare data at baseline and 6 months. ** P < 0.01

Oral semaglutide significantly decreased HbA1c and body weight during the 6-month study period (Fig. 2, Supplementary Table 1). These effects were similar in subjects who had switched from a DPP-4i, who were the majority of the subjects in the present study (Supplementary Table 1). These effects were similar in participants in each age bracket (Supplementary Table 2). At baseline, HbA1c was high in most of the subjects and achievement rate of glycemic targets from JDS and JGS were only being met 20.8% (Fig. 3, Supplementary Table 3). After 6 months, HbA1c significantly declined (*P* < 0.001) and many had achieved their glycemic targets (prevalence had changed from 25.0% to 70.8%, *P* < 0.01), whereas the late older subjects using insulin or an insulin secretagogue tended to be more likely to have achieved below glycemic target (*P* = 0.18) (Fig. 3, Supplementary Table 3). In addition, the change in HbA1c during the 6-month period correlated with the baseline HbA1c value and the insulin secretory capacity (Supplementary Table 4).

Although neither the metabolic parameters nor body mass significantly differed at baseline between the two age groups (early; 65–74 *versus* late; ≥ 75), the HDS-R of the late older subgroup was significantly lower (*P* < 0.01) (Supplementary Table 2, Supplementary figure 1) and negatively correlated with age (*r* = −0.57, *P* < 0.01) (Supplementary figure 1). On the other hand, the reduction of HbA1c and body weight showed no significant differences between the age groups (early *versus* late: HbA1c; *P* = 0.66, body weight; *P* = 0.27) (Supplementary figure 2).

### Safety

Table 2 shows the details of the adverse events. Severe adverse events, defined using the CTCAE, did not occur (Supplementary information). Discontinuation of the medication was more frequent in the late older subjects and was for the following reasons: Early; vomiting (3 mg, *n* = 1) and anorexia (3 mg, *n* = 1); Late; vomiting (3 mg, *n* = 1; 7 mg, *n* = 1) and anorexia (7 mg, *n* = 1; 14 mg, *n* = 1). Most of the other adverse events that were reported were gastrointestinal symptoms, and none of the subjects experienced hypoglycemia.

**Table 2:** Breakdown of the adverse events during the study

	All subjects (*n* = 30)	Early (*n* = 15)	Late (*n* = 15)
All adverse events, n (%)	21 (70.0)	11 (73.3)	10 (66.7)
Required discontinuation	6 (20.0)	2 (13.3)	4 (26.7)
All GI events, n (%)	13 (43.3)	9 (60.0)	4 (26.7)
Constipation	1 (3.3)	1 (6.7)	0 (0.0)
Vomiting	3 (10.0)	1 (6.7)	2 (13.3)
Nausea	4 (13.3)	2 (13.3)	2 (13.3)
Diarrhea	3 (10.0)	3 (20.0)	0 (0.0)
Abdominal distension	1 (3.3)	1 (6.7)	0 (0.0)
Dyspepsia	1 (3.3)	1 (6.7)	0 (0.0)
Anorexia, n (%)	11 (36.7)	4 (26.7)	7 (46.7)
Palpitations, n (%)	1 (3.3)	0 (0.0)	1 (6.7)
Tremor, n (%)	1 (3.3)	0 (0.0)	1 (6.7)

Adverse events were investigated in eligible subjects except for that who were excluded by reasons other than the effects of the drug of interest (*n* = 30). Adverse events required discontinuation of medication were following reasons, but all of them graded 1 based on CTCAE (Early; vomiting, *n* = 1; anorexia, *n* = 1, Late; vomiting, *n* = 2; anorexia, *n* = 2)

*CTCAE* Common terminology criteria for adverse events, Early; 65–74 years old, *GI* Gastrointestinal, Late, ≥ 75 years old

### Sample size

Since the sample size might not have been sufficient, we calculated the post hoc power. The overall detection power value under the 5% α error was 1.0 for the change in HbA1c between baseline and 6 months after switching to oral semaglutide (*N* = 24). In each age bracket, the overall detection power value under the 5% α error were 0.999 (65–74 years old, *n* = 13) and 0.999 (≥ 75 years old, *n* = 11).

## Discussion

In the present study, we have evaluated the efficacy and safety of oral semaglutide for older subjects, and compared relevant parameters in subjects of 65–74 years and ≥ 75 years. Although severe adverse events, involving drug discontinuation, were relatively frequent in subjects of ≥ 75 years, we found that oral semaglutide treatment was effective, as shown in previous phase 3 clinical trials [14]. Furthermore, we characterized the medication and cognitive function of these subjects, and given that DPP-4is were the most frequently prescribed drugs at baseline, the study may provide useful information regarding the efficacy of switching from a DPP-4i to an oral GLP-1RA in patients of this age. To the best of our knowledge, this is the first study to characterize the safety and efficacy of oral semaglutide in older patients with T2D in a real-world clinical setting.

The PIONEER clinical trials showed the efficacy of oral semaglutide in subjects from around the world [20]. Additional analyses for older Japanese subjects with T2D were conducted in PIONEER 9 and 10 [14, 21, 22]. Japan is one of the countries with a rapidly aging population, specifically, 29.1% of the population was ≥ 65 years old and 15.5% was ≥ 75 years old in 2022. The baseline age of the previous sub-analysis was 65 years old and more precise classification was desirable to capture the real-world setting [14]. Here, we compared the efficacy of semaglutide in subgroups of subjects of 65–74 years and ≥ 75 years to identify any differences between these sections of the older population [23]. As in PIONEER 9 and 10, we found that semaglutide reduced HbA1c and body weight in both age groups (Supplementary Table 2).

Because older patients can be vulnerable to adverse effects of drugs [24], it is important to clarify whether new therapeutics have such effects in this population. A sub-analysis of the PIONEER trials showed that older patients of ≥ 65 years had a higher discontinuation rate of oral semaglutide than younger subjects [14]. In the present study, nearly 80% of the older patients continued the medication for the full 6 months, but discontinuations were slightly more frequent in those who were ≥ 75 years old (*n* = 4, 26.7%) than in those of 65–74 years of age (*n* = 2, 13.3%), and these most frequently reason were due to gastrointestinal symptoms. Notably, two-thirds of the dropouts occurred at the starting dose (3 mg). In addition, the subjects lost approximately 6% of their total body mass during the study, despite having normal BMIs, which implies that their muscle mass may have also been reduced (Supplementary Tables 1 and 2). Therefore, careful explanations should be provided when oral semaglutide therapy is commenced, especially in very aged patients, and clinicians should be aware that the glycemic targets for older patients should be determined according to their age, activity level, and the use of other medication [18]. In our results, oral semaglutide use was associated with a 70.8% achievement rate with respect to glycemic targets, but also undesirably low HbA1c values in 20.8% of the participants (Fig. 3, Supplementary Table 3). In a sub-analysis of PIONEER 10, 2.5% of subjects administered oral semaglutide experienced symptomatic hypoglycemic episodes, and most of these were also administering sulfonylureas; however, their age and renal function were unknown [25]. Although hypoglycemic symptoms were not reported in the present study, the identities of any concomitant medications, and especially of anti-hyperglycemic agents that can be associated with severe hypoglycemia, should be taken into account when the use of semaglutide is considered for patients of ≥ 75 years.

DPP-4is are the most frequently prescribed anti-hyperglycemic agents in Japan, because of their convinced safety and effectiveness [26]. In addition, the algorithm that was recently published by the JDS and JGS recommend their use primarily for patients who do not have obesity, and in fact DPP-4is tend to be prescribed more frequently in older patients [26, 27]. This is reflected in over 90% of the participants administering a DPP-4i at the start of the present study, precisely reflecting the trends of prescription in our country. Importantly, however, the participants in the PIONEER phase 3 trials were not administering a DPP-4i at the start [20–22]. Collectively, the present study provides important insights into the clinical usefulness of oral semaglutide, in particular after switching from a DPP-4i, in older patients.

The complexity of the method of administration is one of the problems associated with oral semaglutide use [13], and this might be a challenge for patients with cognitive impairment. In fact, cognitive impairment does not significantly affect adherence to oral medications or self-care in patients with T2D, rather injections including insulin might be a problem [28]. However, the circulating semaglutide concentration following administration is more variable following oral administration than subcutaneous injection, which might reflect poorer absorption [29]. Nevertheless, the established potent antihyperglycemic effect of oral semaglutide was replicated in the present study, even though it was of older subjects. Cognitive function declined with increasing age, whereas did not correlate with improvements of HbA1c in the present study (Supplementary figure 1, Supplementary Table 4). Although, it is worthy to note that subjects suffering severe cognitive impairment were not involved in this study. Instead, the improvement in HbA1c correlated with CPR and CPI, suggesting that the level of residual beta-cell function is associated with the efficacy of oral semaglutide treatment, which has been shown for other GLP-1RAs (Supplementary Table 4) [30–32].

The strengths of the present study were that we exclusively studied older patients, that we assessed the efficacy and safety of the drug in a real-world clinical setting, and that the cognitive function of the participants was also evaluated. We have also confirmed the trends in prescribing practice in Japan and demonstrated the effectiveness of switching from a DPP-4i to oral semaglutide. However, there were also several limitations. First, it was a single-arm retrospective observational study with a small sample size. Second, the participants were exclusively Japanese, which limits the generalizability of the findings. Third, the effects on muscle and the sarcopenia associated with weight loss have not been adequately investigated Fourth, because the study was conducted of outpatients under real-world clinical conditions, it was not possible to accurately assess whether the actual medication regimen and the self-administration of the medications were adequate or to what extent the subjects were compliant. Finally, the subjects were older, but had relatively well-preserved cognitive function. Therefore, further studies should be conducted in the next future, including larger, two-arm, prospective studies, and studies of participants with more marked cognitive impairment.

## Conclusions

In conclusion, we have demonstrated the efficacy of oral semaglutide in older subjects in a real-world clinical setting. The frequency of adverse events for older subjects was similar to that in the clinical trial, however, discontinuation of medication and risk for potential hypoglycemia were more frequent in late older subjects. Therefore, it is important that clinicians provide careful explanations and appropriate concomitant treatments, while considering the risk/benefit ratio. Although appropriate treatment goals should be set for older patients with diabetes, paying attention to their individual characteristics, oral semaglutide may be an effective means of achieving these goals in older patients as a whole.

### Supplementary Information

Supplementary Material 1: Supplementary figure 1. Relationship between age and cognitive function. Supplementary figure 2. Changes in HbA1c and body weight during the study in each age group.

Supplementary Material 2.

## Data Availability

The data that support the findings of this study are available on request from the corresponding author. The data have not been made publicly available owing to privacy or ethical restrictions.

## Abbreviations

BMI: Body mass index
CTCAE: The Common Terminology Criteria for Adverse Events
CPI: C-peptide index
CPR: C-peptide
DASC-8: The Dementia Assessment Sheet for Community-based Integrated Care System 8-items
DPP-4is: Dipeptidyl peptidase-4 inhibitors
GLP-1RAs: Glucagon-like peptide-1 receptor agonists
HbA1c: Glycated hemoglobin
HDS-R: The Hasegawa Dementia Rating Scale-revised
JDS: The Japan Diabetes Society
JGS: The Japan Geriatrics Society
T2D: Type 2 diabetes
UMIN: The University Hospital Medical Information Network
