# Supplementary material for: Efficacy and safety of oral semaglutide in older patients with type 2 diabetes: a retrospective observational study (the OTARU-SEMA study)
Source: BMC Endocr Disord. 2024 Jul 24;24:124. doi: 10.1186/s12902-024-01658-6 (PMC11267784; doi:10.1186/s12902-024-01658-6)
Supplement: Supplementary file 1 — Supplementary Material 1: Supplementary figure 1. Relationship between age and cognitive function. Supplementary figure 2. Changes in HbA1c and body weight during the study in each age group. [file 12902_2024_1658_MOESM1_ESM.zip › Supplementary Information.docx]

**Supplementary information**

The other exclusion criteria were as follows: 1) a history of hypersensitivity to semaglutide components, 2) diabetic ketosis/coma or pre-coma, 3) unsuitability for another reason.

All adverse events were evaluated by common terminology criteria for adverse events (CTCAE) in this study (1). Severe adverse events were defined as following; 1) deadly, 2) life- threatening, 3) hospitalization or prolongation of existing hospitalization indicated, 4) permanent or extreme disability or dysfunction occurred consequently, 5) causing congenital, familial and genetic disorders. All discontinuations of medication were decided after evaluating the grade of adverse events based on the CTCAE. Even if the symptoms were mild on grading, the medication discontinued if the patient so desired.

Reference

1. Common Terminology Criteria for Adverse Events (CTCAE) Version 5. Published: November 27. US Department of Health and Human Services, National Institutes of Health, National Cancer Institute.

Supplementary figure 1. Relationship between age and cognitive function

(a) HDS-R score, according to age group. HDS-R score in 65–74-year-old and ≥75-year-old participants. Bars represent mean (95% confidence interval). Comparison was made using the unpaired *t*-test. ** *P*<0.01, *** *P*<0.001.
(b) Pearson correlation between age and HDS-R score. r=−0.57, *P*<0.01.

HDS-R, the Hasegawa Dementia Rating Scale-revised.

Supplementary figure 2. Changes in HbA1c and body weight during the study in each age group

(a) Change in HbA1c. (b) Change in body weight

There were no significant differences between the 65–74-year-olds and the ≥75-year-olds. Bars represent mean (95% confidence interval). Comparisons were made using the unpaired *t*-test.
