# Supplementary figures and images for: Efficacy and safety of oral semaglutide in older patients with type 2 diabetes: a retrospective observational study (the OTARU-SEMA study)
Source: BMC Endocr Disord. 2024 Jul 24;24:124. doi: 10.1186/s12902-024-01658-6 (PMC11267784; doi:10.1186/s12902-024-01658-6)

## Slide 1
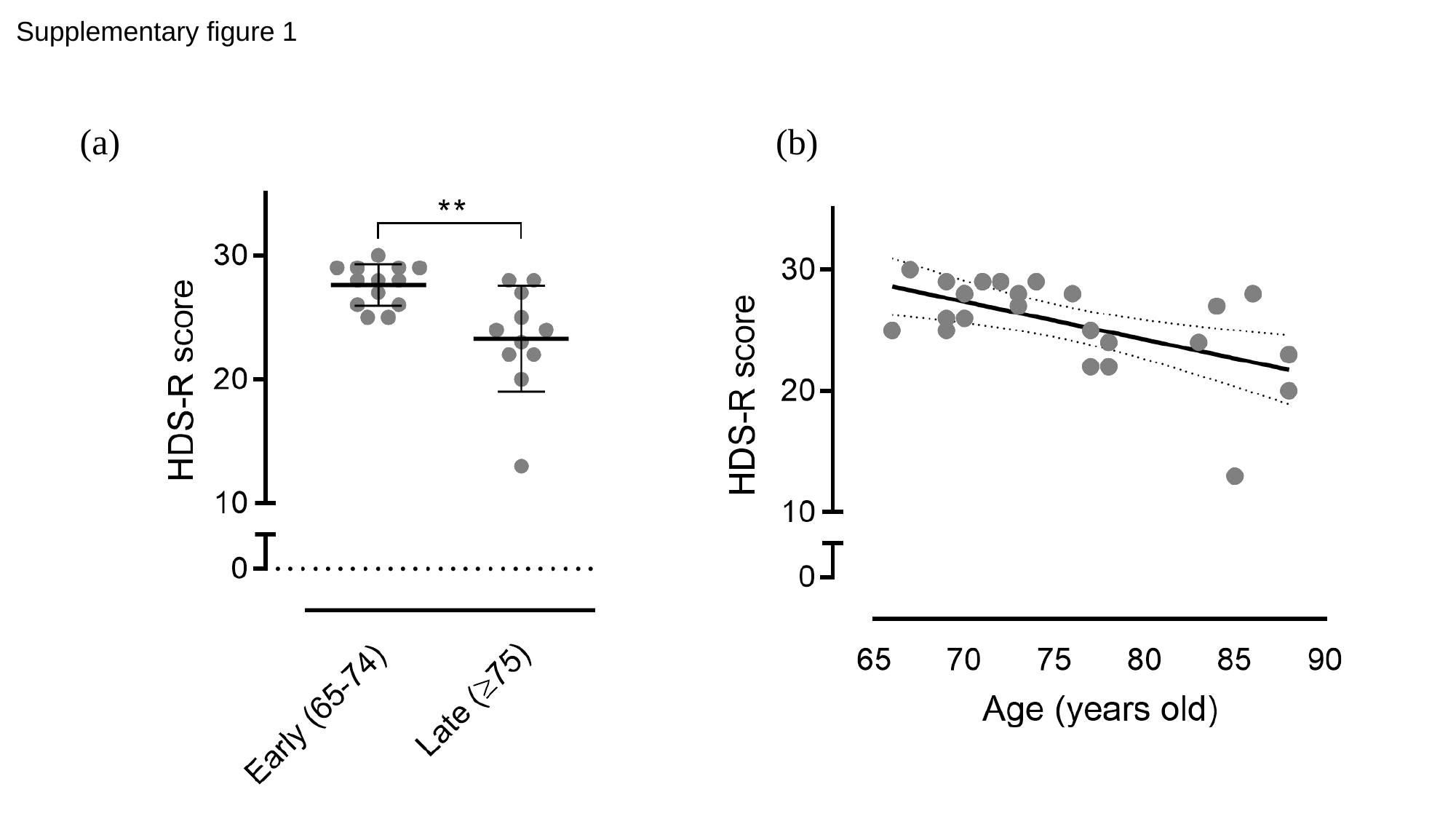

Supplementary figure 1
(a)
(b)

Supplement: Supplementary file 1 — Supplementary Material 1: Supplementary figure 1. Relationship between age and cognitive function. Supplementary figure 2. Changes in HbA1c and body weight during the study in each age group. [file 12902_2024_1658_MOESM1_ESM.zip › Supple_figure 1.pptx]

## Slide 1
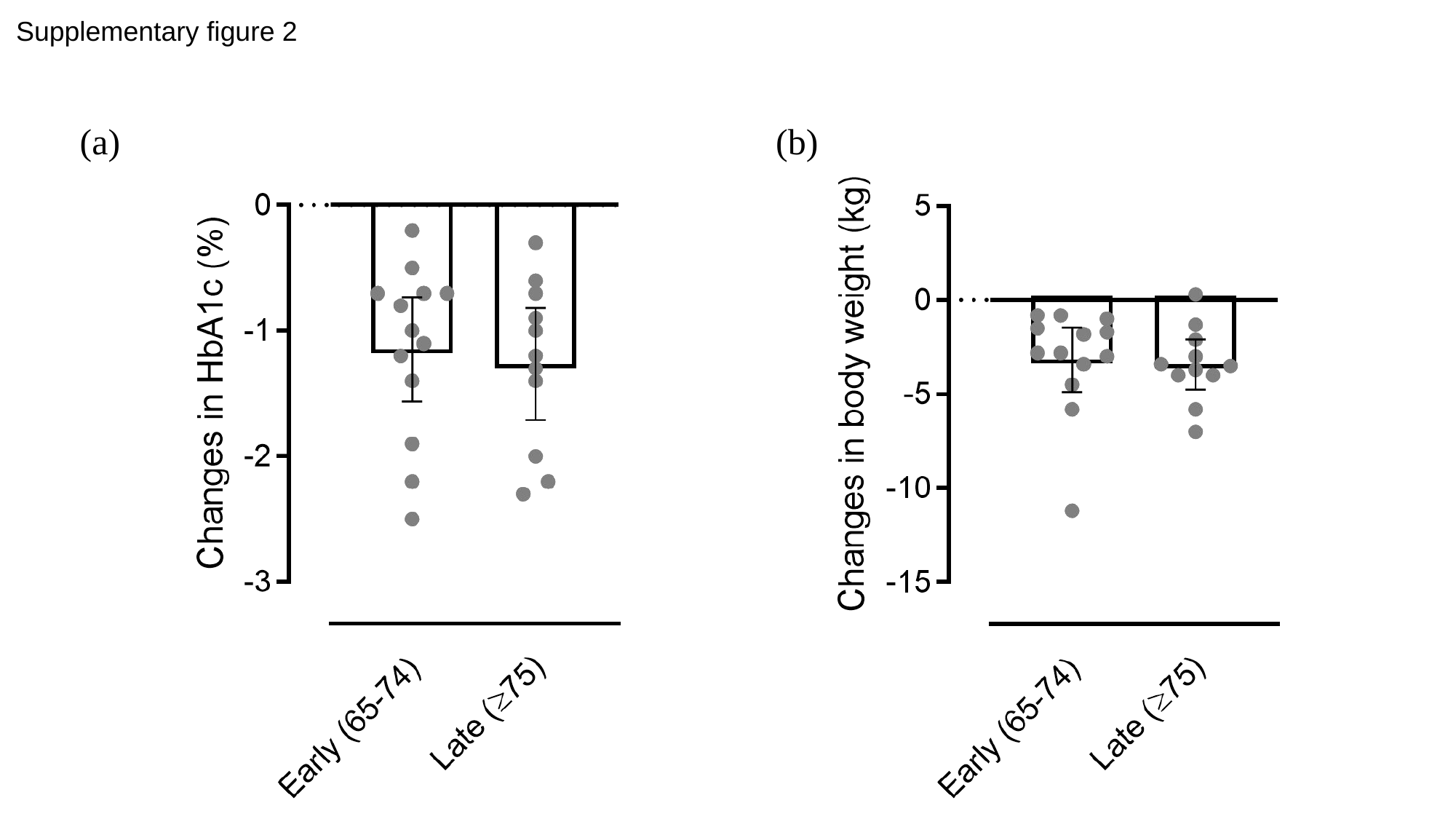

Supplementary figure 2
(a)
(b)

Supplement: Supplementary file 1 — Supplementary Material 1: Supplementary figure 1. Relationship between age and cognitive function. Supplementary figure 2. Changes in HbA1c and body weight during the study in each age group. [file 12902_2024_1658_MOESM1_ESM.zip › Supple_figure 2.pptx]
