# Supplementary material for: Efficacy and safety of oral semaglutide in older patients with type 2 diabetes: a retrospective observational study (the OTARU-SEMA study)
Source: BMC Endocr Disord. 2024 Jul 24;24:124. doi: 10.1186/s12902-024-01658-6 (PMC11267784; doi:10.1186/s12902-024-01658-6)
Supplement: Supplementary file 2 — Supplementary Material 2. [file 12902_2024_1658_MOESM2_ESM.docx]

Supplementary table 1. Changes in metabolic parameters during 6 months.

| Variables | baseline | 6 months | *P* value |
| --- | --- | --- | --- |
| Body weight (kg) | 57.4 ± 11.5 | 54.1 ± 11.9 | <0.01 |
| BMI (kg/m^2^) | 23.7 ± 3.4 | 22.3 ± 3.7 | <0.01 |
| HbA1c (mmol/mol) | 63.1 ± 8.5 | 50.0 ± 5.9 | <0.01 |
| AST (IU/L) | 23.8 ± 9.2 | 24.5 ± 10.1 | 0.56 |
| ALT (IU/L) | 20.5 ± 8.6 | 20.5 ± 9.7 | 0.95 |
| Creatinine (µmol/L) | 79.2 ± 26.3 | 80.1 ± 23.5 | 0.60 |
| eGFR (mL/min/1.73m^2^) | 59.7 ± 17.4 | 58.0 ± 15.8 | 0.20 |
| Switching from DPP4-I (N=22) | |  |  |
| Body weight (kg) | 57.7 ± 11.8 | 54.5 ± 12.1 | <0.01 |
| BMI (kg/m^2^) | 23.8 ± 3.5 | 22.5 ± 3.7 | <0.01 |
| HbA1c (mmol/mol) | 63.4 ± 8.6 | 50.7 ± 5.6 | <0.01 |
| AST (IU/L) | 24.3 ± 9.4 | 25.0 ± 10.3 | 0.59 |
| ALT (IU/L) | 20.8 ± 8.9 | 20.7 ± 10.0 | 0.95 |
| Creatinine (µmol/L) | 79.6 ± 27.1 | 80.5 ± 24.0 | 0.64 |
| eGFR (mL/min/1.73m^2^) | 59.6 ± 18.1 | 57.8 ± 16.4 | 0.23 |

Values are expressed as mean ± SD or median (interquartile range). Changes of metabolic parameters in subjects with DPP4-inhibitors as pre-treatments during 6 months.*P*-values were obtained by Student’s *t*-test. ALT, alanine aminotransferase; AST, aspartate aminotransferase; BMI, body mass index; DPP-4i, dipeptidyl peptidase-4 inhibitor; eGFR, estimated glomerular filtration rate; HbA1c, glycated hemoglobin.

Supplementary table 2. Baseline and 6 months after parameters by age bracket.

|  | Early (65-75 years old; n=13) | | | Late (≥ 75 years old; n=11) | | |
| --- | --- | --- | --- | --- | --- | --- |
| Variables | Baseline | 6 months | *P* value | Baseline | 6 months | *P* value |
| Age (years) | 70.2 ± 2.4 ^§^ |  |  | 81.8± 4.7 ^§^ |  |  |
| Female sex (n) | 7 |  |  | 5 |  |  |
| Height (cm) | 156.1± 7.1 |  |  | 154.2 ± 10.5 |  |  |
| Diabetes duration (years) | 14.4 ± 11.9 |  |  | 21.7 ± 12.8 ^¶^ |  |  |
| CPR (nmol/L) | 0.7 ± 0.3 |  |  | 0.6 ± 0.2 ^¶^ |  |  |
| UACR (mg/gCr) | 33.9 [22.0, 58.7] |  |  | 39.8 [22.4, 168.5] |  |  |
| DASC-8 | 8.0 [8.0, 9.0] |  |  | 9.0 [8.0, 14.0] |  |  |
| BADL | 3.0 [3.0, 3.0] |  |  | 3.0 [3.0, 3.0] |  |  |
| IADL | 3.0 [3.0, 3.0] |  |  | 3.0 [3.0, 9.0] |  |  |
| HDS-R | 27.6 ± 1.7 ^§^ |  |  | 23.3 ± 4.3 ^§^ |  |  |
| Body weight (kg) | 58.5 ± 9.6 | 55.4 ± 10.4 | <0.01 | 56.0 ± 13.9 | 52.6 ± 13.9 | <0.01 |
| %TWL (%) |  | -5.6 ± 4.8 |  |  | -6.3 ± 4.1 |  |
| BMI (kg/m^2^) | 24.0 ± 3.3 | 22.7 ± 3.6 | <0.01 | 23.4 ± 3.7 | 21.9 ± 3.9 | <0.01 |
| HbA1c (mmol/mol) | 62.0 ± 8.7 | 49.5 ± 6.3 | <0.01 | 64.4 ± 8.6 | 50.6 ± 5.5 | <0.01 |
| AST (IU/L) | 23.5 ± 9.8 | 22.7 ± 11.1 | 0.70 | 24.1 ± 8.8 | 26.5 ± 8.8 | 0.07 |
| ALT (IU/L) | 19.4 ± 7.8 | 19.6 ± 11.4 | 0.91 | 21.9 ± 9.7 | 21.5 ± 7.5 | 0.74 |
| Cr (µmol/L) | 84.5 ± 30.6 | 85.1 ± 28.3 | 0.82 | 73.0 ± 19.9 | 74.3 ± 15.4 | 0.52 |
| eGFR (mL/min/1.73m^2^) | 56.0 ± 13.9 | 55.5 ± 15.3 | 0.75 | 64.1 ± 20.6 | 61.0 ± 16.6 | 0.15 |
| The proportion of comorbidities (n, %) | |  |  |  |  |  |
| Hypertension | 11 (84.6) |  |  | 11 (100.0) |  |  |
| Dyslipidemia | 11 (84.6) |  |  | 7 (63.6) |  |  |
| Chronic kidney diseases | 8 (61.5) |  |  | 5 (45.5) |  |  |
| Cardiovascular disease | 4 (30.8) |  |  | 2 (18.2) |  |  |
| The proportion of anti-diabetic agent (n, %) | |  |  |  |  |  |
| The number of drugs | 3.0 [2.5, 3.5] | 3.0 [2.0, 3.5] | 0.17 | 2.0 [2.0, 2.0] | 2.0 [1.0, 2.0] | 0.34 |
| Biguanide | 9 (69.2) | 10 (76.9) |  | 3 (27.2) | 3 (27.2) |  |
| DPP-4i | 11 (84.6) | 0 (0.0) |  | 11 (100.0) | 0 (0.0) |  |
| SU | 2 (15.4) | 2 (15.4) |  | 4 (36.4) | 3 (27.2) |  |
| Glinides | 2 (15.4) | 1 (7.7) |  | 2 (18.2) | 2 (18.2) |  |
| Thiazolidine | 0 (0.0) | 0 (0.0) |  | 1 (9.1) | 1 (9.1) |  |
| α-GI | 1 (7.7) | 1 (7.7) |  | 0 (0.0) | 0 (0.0) |  |
| SGLT2i | 11 (84.6) | 11 (84.6) |  | 0 (0.0) | 0 (0.0) |  |
| Insulin | 1 (7.7) | 1 (7.7) |  | 1 (9.1) | 1 (9.1) |  |
| The proportion of antihypertensive agent (n, %) | |  |  |  |  |  |
| The number of drugs | 1.0 [1.0, 2.0] |  |  | 2.0 [1.0, 2.0] |  |  |
| ARB/ACE inhibitors | 7 (53.8) |  |  | 7 (63.6) |  |  |
| CCB | 7 (53.8) |  |  | 6 |  |  |
| Diuretics | 2 (15.4) |  |  | 0 |  |  |
| The proportion of lipid-lowering agent (n, %) | |  |  |  |  |  |
| The number of drugs | 1.0 [1.0, 2.0] |  |  | 1.0 [0.0, 1.0] |  |  |
| Statins | 9 (69.2) |  |  | 7 (63.6) |  |  |
| Fibrates | 2 (15.4) |  |  | 0 (0.0) |  |  |
| Ezetimibe | 4 (30.8) |  |  | 1 (9.1) |  |  |

Values are expressed as mean ± SD or median (interquartile range). ¶The values were calculated except one subject for missing data. §Analysis of variance showed a significant difference at baseline between early- and late- groups. ACE, angiotensin-converting enzyme; α-GI, alpha glucosidase inhibitors; ALT, alanine aminotransferase; AST, aspartate aminotransferase; ARB, angiotensin receptor blocker; BADL, basic activities of daily living; BMI, body mass index; CCB, calcium channel blocker; CPR, C-peptide; Cr, creatinine; DASC-8, dementia assessment sheet for community-based integrated care system 8-items; DPP-4i, dipeptidyl peptidase-4 inhibitor; eGFR, estimated glomerular filtration rate; HbA1c, glycated hemoglobin; HDS-R, Hasegawa dementia rating scale-revised; IADL, instrumental activities of daily living; %TWL, percent of total weight loss; SGLT2i, sodium-glucose cotransporter 2 inhibitor; SU, sulfonylurea; UACR, Urine albumin to creatinine ratio.

Supplementary table 3. Achievement rate of glycemic targets for elderly patients with diabetes.

|  |  | Early (n=13) | Late (n=11) |
| --- | --- | --- | --- |
| Within the range | Baseline | 2 (15.4%) | 3 (27.3%) |
|  | 6 mo. | 11 (84.6%) *** | 6 (54.5%) |
| Above glycemic target | Baseline | 11 (84.6%) | 7 (63.6%) |
|  | 6 mo. | 2 (15.4%) *** | 0 (0%) ** |
|  |  | Early (n=7) | Late (n=5) |
| Below glycemic target | Baseline | 0 (0%) | 1 (9.1%) |
|  | 6 mo. | 0 (0%) | 5 (45.5%) |
| The proportion of anti-diabetic agent in below glycemic target subjects at 6 mo. | | | |
| Biguanide |  | NA | 1 (20.0%) |
| DPP-4i |  | NA | 5 (100.0%) |
| SU |  | NA | 2 (40.0%) |
| Glinides |  | NA | 2 (40.0%) |
| Thiazolidine |  | NA | 0 |
| α-GI |  | NA | 0 |
| SGLT2i |  | NA | 0 |
| Insulin |  | NA | 1 (20.0%) |
| Bolus |  | NA | 5 unit |
| Basal |  | NA | 2 unit |

In accordance with glycemic targets for elderly patients with diabetes from Japan Diabetes Society and Japan Geriatrics Society Joint Committee, HbA1c levels were evaluated at baseline and 6 months after by age bracket. Each subject’s background was categorized by DASC-8 score and concomitant drug. With respect to “Below glycemic target”, only subjects who met the criteria were investigated (Early: N=7/13, Late: N=5/11). These numbers evaluated by Fisher's exact test between baseline and 6 months. ** *P* < 0.01, *** *P* < 0.001 *versus* each baseline. α-GI, alpha glucosidase inhibitors; DASC-8, dementia assessment sheet for community-based integrated care system 8-items; DPP-4i,

dipeptidyl peptidase-4 inhibitor; Early, 65-74 years old; Late, ≥75 years old; NA, not applicable; SGLT2i, sodium-glucose cotransporter 2 inhibitor; SU, sulfonylurea.

Supplementary table 4. Simple regression analysis between the change of HbA1c and baseline parameters.

| Variables | r | *P* value |
| --- | --- | --- |
| Age | 0.079 | 0.71 |
| Female sex | 0.090 | 0.68 |
| Height | 0.164 | 0.44 |
| Body weight | 0.092 | 0.67 |
| BMI | −0.028 | 0.90 |
| Diabetes duration^¶^ | −0.179 | 0.41 |
| FPG ^¶^ | 0.052 | 0.82 |
| HbA1c | −0.736 | <0.01 |
| CPR ^¶^ | −0.419 | <0.05 |
| CPI ^¶^ | −0.490 | <0.05 |
| AST | −0.118 | 0.58 |
| ALT | −0.060 | 0.78 |
| Cr | 0.145 | 0.50 |
| eGFR | −0.163 | 0.45 |
| DASC-8 | −0.150 | 0.49 |
| HDS-R | −0.094 | 0.66 |

Regression correlations between ΔHbA1c and preoperative baseline parameters. These values were evaluated by Pearson correlation coefficient. ¶The values were calculated except one subject for missing data.

AST, aspartate aminotransferase; ALT, alanine aminotransferase; BMI, body mass index; CPR, C-peptide; CPI, C-peptide index; Cr, creatinine; DASC-8, dementia assessment sheet for community-based integrated care system 8-items; eGFR, estimated glomerular filtration rate; FPG, fasting plasma glucose; HDS-R, Hasegawa dementia rating scale-revised; HbA1c, glycated hemoglobin.
